# Cancer-Related Fatigue and the Additive Effect of Treatment in the Context of Lymphoma: An Analysis of the Lymphoma Coalition’s 2022 Global Patient Survey

**DOI:** 10.1158/2767-9764.CRC-24-0048

**Published:** 2024-07-01

**Authors:** Steve E. Kalloger, Amanda Watson, Shawn Sajkowski, Lorna Warwick

**Affiliations:** 1 Lymphoma Coalition, Mississauga, Canada.; 2 Department of Pathology and Laboratory Medicine, University of British Columbia, Vancouver, Canada.; 3 School of Population and Public Health, University of British Columbia, Vancouver, Canada.

## Abstract

Cancer-related fatigue (CRF) continues to be a challenging phenomenon that is often underreported and poorly understood. With etiologies in both disease and treatment manifesting as a symptom and a side effect, respectively, CRF is highly incident and presents a significant clinical problem that impacts survivorship. We conducted a survey to ascertain the patient-reported incidence of symptoms and side effects for people with lymphoma or chronic lymphocytic leukemia. We found that CRF was enhanced in those who received more intense therapies that coincided with more aggressive lymphoma subtypes. These data illuminate an unmet need among patients with lymphoma and provide an opportunity to further refine treatment regimens in order to reduce the burden of CRF in this vulnerable population.

**Significance:**

CRF is a highly incident phenomenon in lymphoma that can be ascribed to a combination of causes. We have demonstrated substantial variability across various subtypes of lymphoma and have estimated that nearly half of the reported fatigue comes from treatment. Increased screening for and monitoring of fatigue will yield favorable health-related quality of life that will benefit health technology assessment activities and yield improved outcomes for patients.

## Introduction

The impact of a cancer diagnosis on a patient presents a challenge from multiple aspects. Aside from the disease, treatments add an additional layer of complexity to the cancer journey that manifest in both physical and psychologic detriments (1). A highly prevalent phenomenon among patients with hematologic malignancies is cancer-related fatigue (CRF; ref. 2). The etiology of CRF can be attributed to both the disease and its treatment (3). Previous work has identified various clinical parameters that are associated with fatigue which include decreased performance status, use of pharmaceutical pain management, gastrointestinal upset, lack of sleep, and abnormal hemoglobin and albumin levels (2, 3). Other research has focused upon whether psychologic aspects drive or result in CRF (4). Unfortunately, the directionality remains elusive.

As evidenced by the inclusion of fatigue as a discrete domain of the recently developed QLU-C10D, the importance of CRF in the health-related quality of life of the patient is gaining traction and will have a direct impact on health technology assessment activities (5, 6). Several studies have provided a variety of potential interventions for those suffering from CRF (7). Therefore, it is no longer considered to be an unavoidable consequence of the cancer journey. However, identifying those at risk of fatigue remains elusive. In this study, we sought to explore the prevalence of fatigue as both a symptom and a side effect among a large group of patients with lymphoma or chronic lymphocytic leukemia (CLL). Our goal was to identify the lymphoma subtypes that had the highest levels of fatigue and to gain insights into the attributable fraction that treatment adds to the mosaic of CRF.

## Methods

### Data acquisition

**Figure 1:**
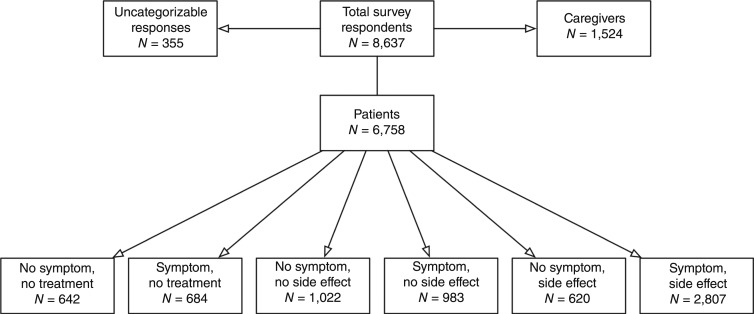
Graphical representation of the respondents to the Lymphoma Coalition’s 2022 Global Patient Survey about the incidence of fatigue as a symptom or side effect. Respondents who did not receive treatment are located on the left side of the graphic.

The Lymphoma Coalition conducted a cross-sectional global survey on patient-reported outcomes and patient-reported experience measures. As part of the survey, patients were asked to report on the incidence of symptoms of their disease and the side effects associated with treatment. Fatigue was a specific domain in both questions. Additionally, patients provided demographic information, their lymphoma subtype (where known), and if they received a medical intervention as part of the care for their lymphoma or CLL. A full factorial approach was used to categorize patients into those who reported fatigue as a symptom, side effect, or both (Fig. 1).

### Statistical modelling

The symptom/side-effect strata for each subtype were subjected to nominal logistic regression to investigate if age and biological sex were significantly associated with fatigue A *P* value of <0.05 was considered statistically significant. All analyses were computed using JMP Pro v17.2 (SAS Institute, Cary NC, USA).

### Estimation of CRF attributable to treatment

The fraction of fatigue that is amplified by treatment (*f*_*SE*_) was estimated using the following equation:

$$f_{\text{SE}}=\frac{C+D}{A+B+C+D}$$ (A)

in which *A* is the number of people who did not report fatigue, *B* those who reported fatigue as a symptom only, *C* those who reported fatigue as a side-effect only, and *D* those who reported fatigue as both a symptom and a side effect.

### Data availability

The data generated in this study are available upon request from the corresponding author.

## Results

Evaluable responses were received from 71 countries, with those from France, the United States, China, Italy, Australia, and the United Kingdom of Great Britain and Northern Ireland accounting for 65% of the cohort. Of the 6,758 survey respondents with evaluable data, 13 subtypes were represented with two categories for other indolent or aggressive lymphomas, respectively. The highest number of responses came from those with CLL (*N* = 1,222), follicular lymphoma (*N* = 1,071), and diffuse large B-cell lymphoma (DLBCL; *N* = 906). Demographics for the symptom/side-effect strata are displayed in Table 1.

**Table 1:** Age and biological sex as reported by survey respondents distributed across the symptom/side-effect strata

Variable	Level	No symptom,no treatment	Symptom, no treatment	No symptom, no side effect	Symptom, no side effect	No symptom, side effect	Symptom, side effect
Age (years)	Mean (SD)	60 (14)	60 (13)	56 (17)	57 (15)	54 (16)	57 (16)
Sex	Female	341 (53%)	643 (65%)	478 (47%)	372 (54%)	356 (57%)	1,756 (63%)
	Male	301 (47%)	340 (35%)	543 (53%)	312 (46%)	264 (43%)	1,047 (37%)
	Intersex	0 (0%)	0 (0%)	0 (0%)	0 (0%)	0 (0%)	1 (0%)
	Prefer not to say	1 (0%)	0 (0%)	0 (0%)	0 (0%)	0 (0%)	3 (0%)

The majority of respondents for all but one subtype category reported fatigue as both a symptom and a side effect of treatment (33%–49%). Supplementary Table S1 provides a summary of the distribution of responses across the symptom/side-effect strata. Approximately 24% of the cohort was excluded from the calculation of treatment-related fatigue because these patients did not receive treatment.

### Covariate associations with fatigue

**Figure 2:**
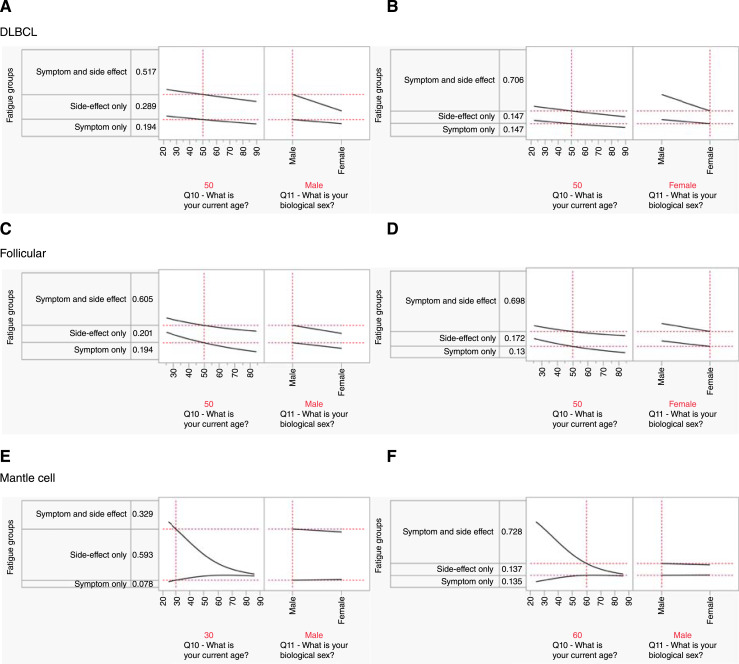
The effect of age and biological sex on the probability of experiencing CRF as a symptom, side effect, or both. **A** and **B,** For patients with DLBCL, the probability of experiencing CRF as both a symptom and side effect increases by a factor of 1.37 for females relative to males given a static age of 50 years. **C** and **D,** Similar but milder phenomenon for patients with follicular lymphoma. Increasing age was also associated with experiencing CRF as both a symptom and side effect. In contrast, the way CRF was experienced by patients with mantle cell lymphoma was not influenced by biological sex but rather age. A 30-year increase in age saw the increase in the probability of experiencing CRF as both a symptom and a side effect by a factor of 2.21 (**E** and **F**).

Utilizing the three strata that received treatment and reported an incidence of fatigue, nominal logistic regression analysis revealed that the combination of the predictors age and biological sex yielded a significant predictive effect for DLBCL, follicular lymphoma, and mantle cell lymphoma (*P* ≤ 0.02). All other subtypes failed to achieve statistical significance for this set of predictors. Examining the predictive effect of each predictor in each subtype demonstrated that females are more likely to experience fatigue as a symptom and side effect relative to males in DLCBL (Fig. 2A and B; *P* < 0.0001). This finding was also shown in follicular lymphoma but just fell short of statistical significance (Fig. 2C and D; *P* = 0.05). Increasing age was also found to be predictive of respondents reporting fatigue as both a symptom and side effect in follicular lymphoma (*P* = 0.03) and mantle cell lymphoma (*P* = 0.004). The effect of age in mantle cell was amplified relative to the other subtypes with respect to the tradeoff between fatigue reported as a side-effect only in younger patients and that reported as both a symptom and side effect in older patients. As an example, a 30-year-old male would have a probability of reporting fatigue as a side-effect only of 0.59, whereas a 60-year-old male would see a probability of reporting fatigue as a side-effect only of 0.13 and an inflation of fatigue as both a symptom and side effect to 0.73. This shows an increase in probability of 0.4 (Fig. 2E and F).

### Amplification of fatigue with treatment

**Figure 3:**
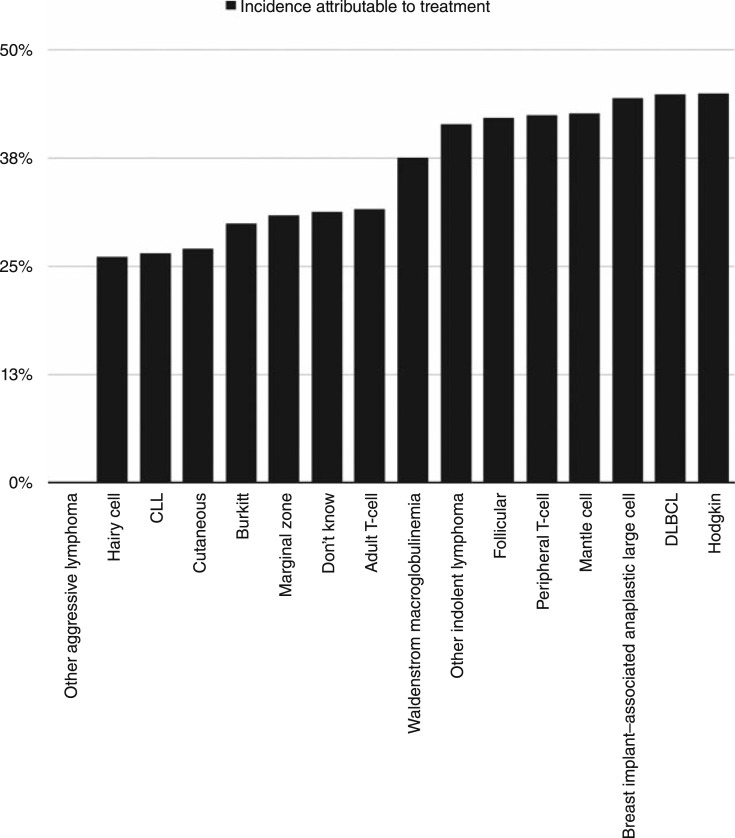
Estimation of the contribution of treatment to CRF, stratified across lymphoma subtypes including CLL. Values are derived from Eq. A in the Methods section.

Across all subtypes, the mean percentage of fatigue attributable to treatment was 34% and ranged from 0% to 45% (Fig. 3). Hodgkin, DLBCL, breast implant–associated anaplastic large-cell, mantle cell, peripheral T-cell, and follicular lymphomas are estimated to have more than 40% of CRF attributed to treatment.

## Discussion

The data in this study indicate that CRF is a highly prevalent phenomenon in lymphoma and CLL. The main issue with regard to the drivers of CRF remains elusive due to the fact that it exists both as a symptom of disease and a side effect of treatment. Although it can be argued that if CRF is a symptom of disease and that an efficacious treatment should relieve CRF as a symptom, the side effects of treatment have the potential to supplant and possibly overshadow any symptom relief. To address this issue more directly, we will be changing our question format in the Lymphoma Coalition’s 2024 Global Patient Survey for symptoms and side effects to scalar measures, which may help identify if CRF intensifies or declines with treatment.

Surprisingly, lymphoma subtypes that are more aggressive tended to have higher levels of CRF both as a symptom and as a side effect. This gives credence to the theory that those who are experiencing CRF may be predisposed to experiencing CRF as a side effect. Although the exact etiology of CRF remains a subject of debate, it undoubtedly encompasses both biological and psychologic causal mechanisms. Our data have demonstrated that females tend to experience CRF as both a symptom and side effect more frequently and that increasing age is a predictor of the same in some lymphoma subtypes. Although these findings reflect those found in previous studies, the majority of lymphoma subtypes represented in our study suggest that other causal mechanisms are at play and clearly illustrate the importance of accounting for subtype in patient-reported outcomes/patient-reported experience measures–based studies. A limitation of this study is related to the argument that CRF attributed to treatment may be transitory in nature. Due to our study design, we are unable to follow patients longitudinally. However, a study by Rüffer and colleagues (8) has demonstrated that patients with Hodgkin lymphoma, who have encountered treatment, continue to experience significant CRF several years after treatment while in remission. This finding provides evidence that CRF is unlikely to be a transitory phenomenon in Hodgkin lymphoma, and further research would serve to confirm if this is the case for other subtypes.

With approximately two thirds of our survey respondents reporting CRF, it can be concluded that CRF is a significant unmet need that impacts survivorship for those with lymphoma or CLL. The impact of CRF negatively affects several physical and psychologic domains quantified in health-related quality of life studies (9, 10). In prospective studies evaluating the novel therapeutics in lymphoma, it will be imperative to identify and provide treatment to those who experience CRF. The guidelines provided by the National Comprehensive Cancer Network are a place to start, as they recognize three discrete stages of the cancer journey: treatment, recovery, and end of life (11). Although the suggested interventions differ only slightly between the stages, they have all proven to be effective. Implementation of these guidelines with continued additions and refinements will not only provide the optimal generation of evidence for health technology assessment activities but also improve the lives of patients with the disease.

In summary, the data contained in this study suggest that CRF should be continuously assessed in patients with lymphoma and CLL and that once identified, they should be treated with one or more of many efficacious options (12). We advocate for the expansion of the definition of precision medicine to include not only the provision of optimal anticancer therapy to the right patients but to also ensure that patients receive the appropriate supportive care. To do so will ensure that patients with lymphoma have the best experience possible during their treatment, recovery, and rehabilitation.

## Supplementary Material

Table S1 Full factorial of symptom and side-effect frequencies across subtypes.
