# Supplementary material for: Cancer-Related Fatigue and the Additive Effect of Treatment in the Context of Lymphoma: An Analysis of the Lymphoma Coalition’s 2022 Global Patient Survey
Source: Cancer Res Commun. 2024 Jul 1;4(7):1620–4. doi: 10.1158/2767-9764.CRC-24-0048 (PMC11195524; doi:10.1158/2767-9764.CRC-24-0048)
Supplement: Table S1 — Full factorial of symptom and side-effect frequencies across subtypes. [file crc-24-0048_table_s1_supps1.pdf]

Kalloger et al. Table S1

Subtype	No Symptom, No Side-effect	Symptom, No Side-effect	No Symptom, Side-effect	Symptom, Side-effect	Total Treated	Incidence attributable to treatment
**Other Aggressive Lymphoma**	32.10%	0.00%	0.00%	0.00%	32.10%	0%
**Chronic Lymphocytic Leukemia**	0.00%	9.25%	3.44%	29.21%	41.9%	26.51%
**Cutaneous**	10.47%	9.22%	4.81%	33.67%	58.17%	27.08%
**Adult T-cell**	14.90%	7.41%	8.64%	38.27%	69.22%	31.67%
**Hairy Cell**	13.54%	23.53%	0.00%	35.29%	72.36%	26.09%
**Waldenstrom's Macroglobulinemia**	11.76%	14.76%	3.69%	44.64%	74.85%	37.61%
**DLBCL**	0.00%	12.25%	15.01%	48.79%	76.05%	44.88%
**Don't know**	29.31%	7.69%	5.77%	33.65%	76.42%	31.30%
**Peripheral T-cell**	10.03%	8.70%	12.50%	47.28%	78.51%	42.47%
**Burkitt's**	16.65%	17.24%	8.62%	37.93%	80.44%	30.00%
**Marginal Zone**	28.06%	9.96%	8.66%	33.77%	80.45%	30.91%
**Other Indolent Lymphoma**	18.67%	7.84%	12.01%	44.12%	82.64%	41.49%
**Follicular**	18.61%	8.22%	12.14%	44.26%	83.23%	42.12%
**Mantle Cell**	19.57%	8.89%	8.89%	49.33%	86.68%	42.67%
**Hodgkin**	15.44%	8.73%	14.24%	48.56%	86.97%	44.98%
**Breast Implant Associated Anaplastic Large Cell**	12.50%	23.08%	15.38%	46.15%	97.11%	44.44%
